# Adolescents’ attitudes, habits, identity and social support in relation to physical activity after the COVID-19 pandemic

**DOI:** 10.1038/s41598-024-60548-y

**Published:** 2024-05-14

**Authors:** Ivana Matteucci, Mario Corsi

**Affiliations:** Department of Communication Sciences, International Studies and Humanities, University of Urbino Carlo Bo, 61029 Urbino, PU Italy

**Keywords:** Adolescents, COVID-19, Cognitive processes, Social support, Physical activity, Psychology, Health care

## Abstract

This study focuses on adolescents’ cognitive processes, behaviors and social support (SS) as they relate to physical activity (PA) before and after the pandemic. The aims of the study were: (1) to investigate the changes in adolescents’ engagement in moderate and vigorous physical activity (MVPA), and examine the changes in PA-related attitudes and behaviors before and after the COVID-19 pandemic; (2) to analyze the correlations between the significant changes that were found, PA engagement, and SS. The survey targeted third-year middle school students of Italian nationality, attending male and female mixed classes, residents in urban, periphery and sub-urban areas, living in families with different incomes, and different habits of engaging in PA. A longitudinal study was developed using a standardized questionnaire. The questionnaire was administered in April–May 2023 to a sample of 952 students aged 11/14 residing in the Marche region in Central Italy. Increasing values were found in the post-COVID-19 phase for all the cognitive processes and attitudes, in particular, those regarding habits (0.66 vs 0.50, + 32%) and identity (0.70 vs 0.55, + 27%) related to PA. Significant correlations were found between these values and VPA engagement and between the values of the same indicators and SS (*p* < 0.01). The strongest relationship was found with the dimension of identity (r = 0.51; r = 054).

## Introduction

Several immediate changes in personal outlooks, social dynamics, and community settings shaped adolescent experiences in the realm of sport and physical activity (PA) during the COVID-19 pandemic, yet questions remain regarding short-, and long-term developmental outcomes. To gain a deeper understanding of the impact of this crisis, it is crucial for researchers and practitioners to examine its effects on youth on various levels^1^. On a personal level, it is important to focus on young athletes and their development into adulthood, seeking to understand their perceived attitudes, habits and opportunities related to PA. On a social level, it is necessary to determine how the main key stakeholders are striving to maintain quality relationships with young people. On a global level, the findings of the literature review on COVID-19 and PA, indicated a decrease in PA among adolescents, which was associated with altered well-being levels, eating habits, and leisure activities^2–5^. These reductions in PA due to the COVID-19 pandemic require public interventions to rapidly restore pre-pandemic PA levels, so that young people are not denied the well-known physical and mental health benefits and psychosocial-relational well-being associated with regular PA^6,7^.

Schöttl et al.^8^ reported that despite the short-term negative effects of COVID-19 restrictions on PA during lockdowns, most subjects returned to original PA levels when COVID-19 restrictions were relaxed. These findings appear to bode well for post-pandemic PA habits. However, as shown by the comparison between alpine regions examined in the same study, particularly severe COVID-19 measures appear to have reduced PA, with potential adverse health effects. Many recommendations regarding the resumption of sport and PA after the pandemic have been issued by authoritative institutions (e.g. IOC—International Olympic Committee)^9^ and by scientists who underline the need for public policies that help restore PA and athletic activity levels, especially among young people^10^. A rapid review aiming to show wheteher COVID-19 ha impacted people’s PA and sedentary behavior, suggested an overall negative impact of COVID-19 on PA, with differential effects across different sub-populations. Significant knowledge gaps were also found in the roles of social and physical attributes that could promote physical activity during pandemics with reduced safety risks^11^.

Habit formation interventions and habit interruption have been demostrated to influence PA (fostering or reducing it) during adolescence^12,13^. Habit has been characterized as an important predictor of changes in MVPA in pandemic restrictions^14^, and it has been seen as salient predictor of adolescent PA^15^. Kwan et al.^15^ also found that coping and identity were significantly associated with MVPA among adolescents before the pandemic; these variables were not found to predict MVPA in the middle phase of the pandemic. In a study among young Chinese children and adolescents Jiao et al. demonstrated the usefulness of cultivating resilience through PA against stress and psychological distress caused by the COVID-19 pandemic^16^.

In a model developed in order to define the objectives of mastery and performance with respect to PA in young students attending French schools, various elements were evaluated, including the perception of competence, perceived skills and abilities, the motivational climate in PA. The results revealed that mastery and performance were positively associated with the elements considered^17^.

The Hailey^18^ study on active adults during the COVID-19 pandemic confirmed previous research showing the importance of SS for long-term maintenance of PA engagement, and also reported that such effects extended across contexts. of social restrictions. High SS was associated with a 64% increase in the probability of maintaining PA during the lockdowns and medium SS was associated with a 32% increase in the probability. The findings of a recent study^19^ provided valuable insights into the context of youth PA following the global pandemic and suggested that families, sport clubs and sport organizations need additional resources and tools (e.g., parental support) to support sport recovery and ensure youth well-being in the future.

A study on factors blocking change and those facilitating change in PA in Irish adolescents during the COVID-19^20^, reported benefits deriving from social support (SS), especially parental support, which was shown to curb bad PA habits during the pandemic. In this study, half the adolescents reported doing less PA, a third reported doing the same amount and one in five reported doing more PA than usual when COVID-19 restrictions were in place. Those belonging to the latter group all stated that parental support was a facilitator. In addition, parents who were less capable of supporting their children at home were found to do less PA.

The literature provided sufficient evidence to demonstrate the reduction in PA engagement during the pandemic^21–25^, researchers also reported changes in the health of adolescents during the lockdowns with an increase in psychological and social distress^26–30^. Nevertheless, scientific investigation on cognitive and social processes and PA engagement before and after the pandemic is limited. There is a lack of analysis about habit, attitudes, intention, and identity (which are all elements at the basis of the behavioral change), perceived social support (which strongly influences personal choices), and the actual engagement in physical activity. PA is an important component of adolescent health, since high levels of PA in youth are associated with better physical health, mental health, cognitive functioning, and academic achievement^31–33^. It should be considered that the decline in adolescent PA-related behaviors due to the COVID-19 pandemic might have future health implications, because PA spans from adolescence to adulthood^34,35^.

This study tries to identify the links between the variables considered, in order to recommend useful interventions to promote PA among adolescents during their transition into adulthood. The elements investigated were chosen because they were recognized as having an influence on the adoption of correct behaviours or change in behaviour. In this regard, the M-PAC tool is a complete and validated methodological device useful to analyze the different dimensions with respect to the promotion of PA^36^. In summary, the main aim of the research was to fill the gap in knowledge about the role of personal and social attributes that can influence PA engagement during pandemics, and avoid the decline of PA in post-pandemic phases.

The specific aims of the study were:

1. To investigate the changes in adolescents’ engagement in moderate and vigorous physical activity (MVPA), and examine the changes in PA-related attitudes and behaviors before and after the COVID-19 pandemic;
2. To analyze the correlations between the significant changes that were found, PA engagement, and SS (dependent variables).

Based on the literature review and research aims, the specific objectives of the study were:

- To build an integrated tool capable of assessing:
  - The involvement of adolescents currently engaged in MVPA;
  - The attitudes, perceived capacities and opportunities, intentions, behaviors, habits and identity (M-PAC dimensions) of adolescents in relation to PA in both the current post-pandemic and pre-pandemic phases;
  - The SS perceived by adolescents in both the current post-pandemic and pre-pandemic phases.
- Compare the recorded changes of the M-PAC framework and SS dimensions in the pre-pandemic and post-pandemic phases.
- Determine the relationships between:
  - The amount of PA (dependent variable) and the sociographic characteristics of the adolescents;
  - The summary indicators of the M-PAC framework evaluated in the current post-pandemic phase (dependent variable), the amount of PA, and the sociographic characteristics;
  - The summary indicators of SS in the current post-pandemic period (dependent variable), the amount of PA, and the sociographic characteristics of the participants;
  - The summary indicators of SS in the current post-pandemic phase and the summary indicators of the M-PAC framework.

## Methods

In Italy, the law gives the role of protecting those who are the subjects of clinical trials to the Ethics Committees, and assigns to the Ethics Committees the task of reviewing and approving bio-medical and pharmaceutical research protocols. Law n.3, 11 January 2018 and Legislative Decree 52/2019. This study doesn’t involve any intervention or manipulation on human subjects. Approval from ethics committees is not required for this type of study except the authorization to use the data.

In Italy, the protection of individuals with respect to data processing and their communication is regulated by Law 31 December 1996, n. 675 “Protection of persons and other subjects with respect to the processing of personal data”. In this study, all the information and data collection were managed directly by the schools participating in the survey. The research was carried out in accordance with the relevant guidelines and regulations. Informed consent was requested and obtained from all the subjects and/or their legal guardians. The researchers received complete availability of the data for scientific and publication purposes as established in the relevant patnership contract.

### Description of the research instrument

To collect the data necessary to achieve the objective of the research an integrated instrument was built collecting specific tools from the literature.

Table 1 shows the structure of the questionnaire that was used.

**Table 1:**
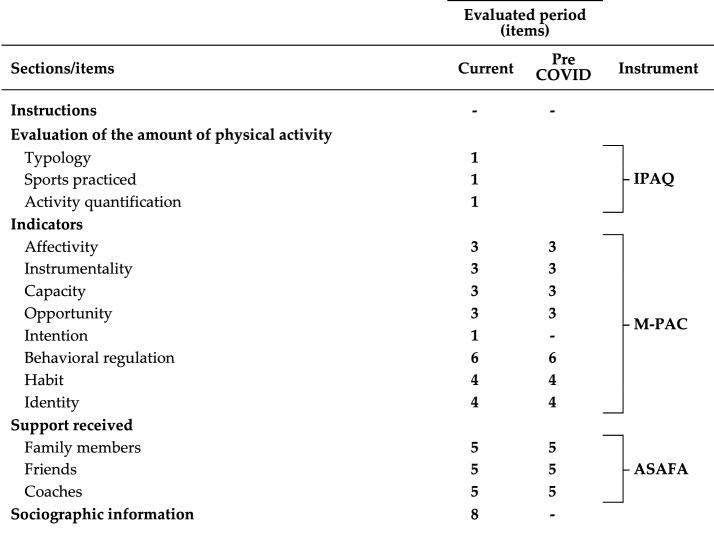
Structure of the questionnaire (skipped questions are excluded).

The first part consists of the IPAQ scale (International Physical Activity Questionnaire)^37,38^ short version, (Available at: https://youthrex.com/wp-content/uploads/2019/10/IPAQ-TM.pdf Accessed on 09/07/2023.) (Available at: https://sensesandsciences.com/download/file/IPAQ_Italiano_BreveAdulti.pdf Accessed on 09/07/2023.) assessing the amount of physical activity currently performed.

The second tool, the M-PAC questionnaire (The Multi-Process Action Control Approach), derives from the M-PAC approach adapted for a population of adolescents and related survey tool^39–41^. The questionnaire was translated into Italian for the study of individual PA and consists of different scales for assessing attitude, perceived capabilities and opportunities, intention, behavioral regulation, habit and identity of adolescents with respect to PA. This tool helps bridge the intention-behavior gap to examine the changes in adolescent PA as a consequence of the COVID-19 pandemic. The M-PAC framework has been employed both to explain and predict PA and to promote it^15,36,42,43^.

According to the M-PAC framework, PA is influenced by three fundamental processes: “reflective processes” which are the deliberate expected consequences of PA execution and behavioral performance; “regulatory processes” which are the behaviors or cognitions that people deploy to translate their initial intentions into PA-related behavior; and “reflexive processes” which denote the impulsive and automatic processes involved in action behavior. “Reflexive processes” include affective attitudes (expected feelings during PA), instrumental attitudes (expected utility of engagement in PA), perceived opportunity (time and access of engagement in PA), and perceived capability (perceptions of ability to engage in PA). These constructs are considered to be antecedents of intention formation. “Regulatory processes” include goal setting, self-monitoring, action planning, and coping planning, which are collectively classified as behavior regulation. Regarding primary “reflexive processes” we can draw a distinction between habit (routine behavioral action performed by cues) and identity (association with a particular role through self-categorization).

The third specific tool consists of the ASAFA scale (Social Support Scale for Physical Activity in Adolescents)^44^, translated into Italian. This tool refers to the SS received by adolescents in performing PA considering three distinct actors: family members, friends and, for those who practice structured physical activity, the people responsible for their training. Social support is described as the assistance offered, or the resources made available in situations of need by different groups, such as parents, siblings, relatives, friends and others. It can be measured by the individual perceptions of the degree to which interpersonal relationships correspond to certain functions (instrumental/direct, psychological/emotional, instrumental/informational)^45^. In the context of PA, SS is one of the main constructs for theories and models that are used in the study of factors associated with physically active behavior. Indeed, SS is consistently associated with higher levels of PA in adolescents^46,47^ and can be offered through incentives, such as shared practice, transportation and assistance provided by key sources of support, including parents, friends and coaches^48,49^.

Both the M-PAC and AFASA tools contain questions (typically on Likert scales) referring to the period of the survey (post-COVID-19) and to the pre-pandemic (pre-COVID-19) period, with the clear aim of investigating any possible changes that may have occurred in the interim. Certainly, the long interval poses serious problems mainly related to the challenge of remembering past habits, and above all -due to the age of the respondents- to changes that may have occurred in their PA habits, regardless of the pandemic crisis. Despite these potential challenges, and in the absence of other information, the investigation was structured as a longitudinal study, but included a retrospective reconstruction of the first observational period.

The last section of the questionnaire concerns the subjects’ sociographic information.

### Survey design

The survey targeted third-year middle school students in the Marche region in Central Italy. Subjects ranged in age from 11 to 14 years; hence, the target population was equal to 55.711 units (ISTAT 2022 data).

For the choice of the sample size, assuming in the first instance, a simple random sampling, and accepting a maximum estimation error of 5%, a minimum size of about 400 students, corresponding to about 23 classes, is obtained. Subjects were recruited through the Regional School Office (Ufficio Scolastico regionale-USR) for the Marche Region—Coordination for Physical and Sports Education. The Office contacted individual physical education teachers, probing their willingness to participate in the initiative. A total of 43 classes, 952 students, agreed to take part in the study. The teachers were duly informed of the aims of the initiative and provided with detailed explanations regarding both the contents of the questionnaire and its correct completion by their students through two training sessions for teachers. At the request of the teaching staff, a video presentation and explanation of the research was also provided to introduce the questionnaire and explain to students how to complete it properly. The video was made available online to all subjects participating in the survey. (The video is available at: https://drive.google.com/file/d/1uF7cAWp-c1v6JhW6yKch-rEsEb80wswy/view?usp=drive_web. Accessed on 03/12/2023).

### Data collection

The questionnaire was administered in the form of a web survey. The survey, produced in an electronic format using an appropriate IT tool (Google Forms application), was first tested by submitting it to a class in their last year of middle school. It was then made available to the entire sample, with online access via the appropriate URL. All the steps envisaged for completing the questionnaire at school required less than one hour.

During the data collection, which lasted for about two months in the spring of 2023, two teachers informed the Authors that they would be unable to collect data from their classes, reducing the target population to 902 subjects divided among 41 classes.

Subsequent data processing relied on the immediate availability of information in an electronic format (Excel spreadsheet). Some preparatory and other specific processing was carried out using Excel, while the bulk of the processing work was done using the SPSS package.

### Data analysis

It wasn’t possible to proceed with the construction of a sample appropriately stratified on the basis of geographical variables. Nevertheless, the data obtained were re-weighted in order to propose a scenario attributable to the entire regional territory. Considering the five provinces and the same number of classes for the demographic size of the municipalities, 25 types of territory were identified (in reality 21, since no municipality referred to 4 of them), providing the aforementioned reweighting of the sample cases with the relative data.

### Statistical analysis

Data processing involved the construction of many aggregate measures, on which analyzes were conducted aimed at verifying the validity of the hypotheses formulated. The t-test was used for single comparisons between groups, while ANOVA analysis with subsequent Post-Hoc analysis through the Scheffe test was used for multiple comparisons. Prior to this, the measurements were tested for normality using the Kolmogorov–Smirnov test. Subsequent analyzes were “forced” even when normality was not guaranteed, relying on the symmetry and marked unimodality of the score distributions. The effect size (Cohen’s d) was calculated where differences were significant. Further analyzes on the relationships between the aggregate measures were done using Pearson’s correlation coefficient.

## Results

### Participation in the survey

On the date of the information extraction, 683 of the 902 identified subjects had completed the survey, indicating a “gross” participation rate of 75.7%. In fact, a check carried out on the data excluding the classes that had not participated, reduced the effective population to 770 subjects with an actual response rate equal to 88.7%.

### Sociographic variables

The reweighted sample reflects, with roughly equivalent shares, the gender composition of the Marche region (ISTAT data), with a slight overrepresentation of the female population (53.6 vs 51.4%).

Given the specific sample group that was chosen for the study, the age of the respondents (average = 13.3 years) is not very informative and clearly reflects their belonging to classes in the third year of middle school.

The variables of the sociographic framework are interesting. The first provides us with information on the educational background of the parents/family members. It is striking that about a quarter of the subjects did not know this information (25.8%), while the reported percentage of at least one parent holding a college degree appears very generous (40.5%).

Based on the questionnaire, about two out of three parents practice sport in some form (67.2%), while as many as one out of seven is a professional (14.4%). We do not have data to verify the accuracy of either of these findings.

### Participation and measurement of physical activity and sociographic information

The quantities of PA found allow us to investigate the possible presence of different behaviors that may be attributable to the sociographic characteristics of the adolescent sample group. The data were thus subjected to an appropriate analysis (dependent-samples t test and ANOVA analysis), which revealed that in some cases there were statistically significant differences (Table 2). The first thing that emerges is the great variability of the data with values of the standard deviation close to the mean values. This clearly makes comparisons difficult, so we will limit ourselves to commenting only on the significant results. The first of these differences concerns moderate physical activity (MPA), with males (267′) significantly more engaged than females (235′). Regarding vigorous physical activity (VPA), the values recorded in families in which adults engage is PA (351′) exceed those where this does not occur (293′). Furthermore, having a family member who engages in PA professionally, in addition to increasing PA, also reproduces the same gap (405′ vs 316′). It would therefore seem justified to conclude that having an example in the family determines at least imitative behavior.

**Table 2:** Amount of weekly physical activity in relation to the sociographic characteristics of the respondents (n = various). Values 1'.

Items	Physical Activity	
	Moderate (SD)	Vigorous (SD)
Gender	*	
Female	235 (181)	314 (268)
Male	267 (266)	342 (267)
Educational background of parents/family members		
Nobody has a high school diploma	214 (184)	276 (217)
At least one has a high school diploma	251 (201)	350 (290)
At least one college graduate	259 (200)	353 (265)
Sports practiced by parent(s)/family member(s)		*
Yes	250 (201)	351 (276)
No	244 (217)	293 (250)
Sports practiced professionally by parent(s) members		**
Yes	237 (211)	405 (279)
No	250 (199)	316 (263)

*One or more significant comparisons at 0.05 (dependent-samples t test; ANOVA analysis).

**One or more significant comparisons at 0.01 (dependent-samples t test; ANOVA analysis).

In the ANOVA analysis, post-hoc analysis was conducted using Scheffe’s test.

For comparisons that were significant, the effect size (Cohen's d) was calculated,

obtaining values ranging from small (d = 0.15) or less to medium (d = 0.30).

### Control-action framework indicators (M-PAC) and amount of PA

As mentioned in the discussion concerning methodology, section B of the questionnaire represents the first part of its core, applying the M-PAC tool to two distinct moments: current/future and pre-COVID-19. M-PAC itself is highly complex and its construct includes several distinct dimensions, each of which is made up of several measurable items. Once the description for each dimension has been made, the internal coherence of the component items is calculated after transforming the attributes on a Likert scale into the same number of equally spaced numerical values, an operation that is not without risks, but is widely practiced. Once this property has been ascertained or accepted, it is possible to construct a summary measure for the entire dimension by adding up the scores related to the individual items, then proceeding with a subsequent normalization.

All the average values of the summary indicators are summarized so that an immediate comparison between the relative dimensions can be drawn (Table 3). At first glance, it can be noted how the dimensions referring to affectivity, instrumentality, abilities and opportunities, to which we can add intention for the immediate future, constitute on the whole, a block with higher values than those recorded for the other dimensions, namely behavioral regulation, habit and identity. Indeed, the M-PAC tool shows a slight discrepancy between intentions and behavior with higher values for the former.

**Table 3:** M-PAC indicators. Average values (AV), standard deviations ((SD)) and internal consistency (Crombach's Alpha) of summary section indicators in the present or near future and in the pre-COVID-19 period (n = 651).

Sections (Items)	Current/Next 4 weeks		Pre COVID-19	
	Alpha	AV (SD)	Alpha	AV (SD)
Affectivity (3)	0.84	0.78 (0.22)	0.92	0.64 (0.30)
Instrumentality (3)	0.84	0.83 (0.20)	0.90	0.72 (0.26)
Capacity (3)	0.75	0.83 (0.18)	0.85	0.69 (0.25)
Opportunity (3)	0.54	0.68 (0.16)	0.61	0.58 (0.20)
Intention (1)	–	0.83 (0.24)	–	–
Behavioral regulation (4)	0.76	0.57 (0.22)	0.86	0.46 (0.26)
Habit (4)	0.83	0.66 (0.25)	0.87	0.50* (*0.28)
Identity (4)	0.85	0.70 (0.25)	0.87	0.55 (0.29)

According to the findings of the present study, the increases in the normalized indicator values related to adolescents’ PA cognitive processes and habits would seem to corroborate the hypothesis of their intention to adopt more virtuous behaviors in the post-pandemic phase. Indeed, on the basis of what was reported by the participants, there were increases in all indicators of the M-PAC tool in the post-pandemic phase compared to the pre-pandemic phase. The relative increases are clear and the most sizeable are found in the dimensions related to habits (0.66 vs 0.50, + 32%), identity (0.70 vs 0.55, + 27%), and behavioral regulation (0.57 vs 0.46, + 24%), while the smallest increases are found in instrumental attitudes (0.83 vs 0.72, + 15%) and opportunities (0.68 vs 0.58, + 17%). It is therefore clear that the COVID-19 pandemic has changed the life behaviors of adolescents, affecting their habits and attitudes related to PA. However, it is possible that other causes may have also played a role in bringing about such changes.

#### M-PAC indicators, amount of PA and sociographic characteristics

In the description, it can be observed how the availability of summary measures helps in the search for possible links between M-PAC indicators and the sociographic variables that identify the students, to which we added those relating to the amount of reported PA. From a methodological standpoint, the analysis of variance was always used in the case of qualitative sociographic variables, while the calculation of the correlation coefficient was used when both were quantitative. The summary data are illustrated in Table 4, and once again, we only considered the statistically significant indications.

**Table 4:** Comparisons between the summary indicators for the M-PAC dimensions assessed in the current period, the amount of physical activity currently performed and the sociographic characteristics of the respondents (n = various).

Item	M-PAC dimensions							
	Affectivity	Instrumentality	Capacity	Opportunity	Intention	Behavioral	Habit	Identity
Weekly amount of moderate activity	0,04	0,03	0,01	0,00	0,03	0,05	0,18^(^**^)^	0,04
Weekly amount of intense activity	0,32^(^**^)^	0,21^(^**^)^	0,28^(^**^)^	0,16^(^**^)^	0,33^(^**^)^	0,19^(^**^)^	0,30^(^**^)^	0,51^(^**^)^
Gender		*					**	
Female	0.77 (0.24)	0.84 (0.18)	0.83 (0.18)	0.84 (0.15)	0.82 (0.25)	0.57 (0.22)	0.63 (0.25)	0.69 (0.25)
Male	0.80 (0.21)	0.81 (0.21)	0.83 (0.18)	0.84 (0.15)	0.85 (0.23)	0.57 (0.22)	0.69 (0.25)	0.71 (0.24)
Parents’ educational background members		**	**	**	**	**	**	**
Nobody has a high school diploma	0.75 (0.24)	0.73 (0.25)	0.76 (0.21)	0.78 (0.16)	0.77 (0.27)	0.49 (0.26)	0.55 (0.23)	0.57 (0.24)
At least one has a high school diploma	0.81 (0.22)	0.85 (0.16)	0.81 (0.19)	0.83 (0.14)	0.84 (0.24)	0.62 (0.19)	0.70 (0.22)	0.70 (0.23)
At least one has a college degree	0.81 (0.20)	0.85 (0.20)	0.88 (0.15)	0.87 (0.13)	0.88 (0.21)	0.57 (0.22)	0.68 (0.27)	0.76 (0.24)
Sports practiced by parent(s)/family members	*	*	**	*	*			**
Yes	0.80 (0.22)	0.84 (0.20)	0.85 (0.17)	0.85 (0.15)	0.85 (0.24)	0.57 (0.22)	0.68 (0.26)	0.73 (0.24)
No	0.76 (0.22)	0.81 (0.17)	0.79 (0.20)	0.82 (0.15)	0.80 (0.24)	0.61 (0.21)	0.64 (0.23)	0.66 (0.23)
Sports practiced professionally								*
Yes	0.82 (0.20)	0.82 (0.22)	0.85 (0.18)	0.83 (0.16)	0.86 (0.23)	0.60 (0.19)	0.68 (0.24)	0.76 (0.23)
No	0.78 (0.23)	0.84 (0.19)	0.83 (0.18)	0.84 (0.14)	0.84 (0.24)	0.56 (0.22)	0.66 (0.26)	0.70 (0.25)

*One or more significant comparisons at 0.05 (dependent-samples t test; ANOVA analysis).

**One or more significant comparisons at 0.01 (dependent-samples t test; ANOVA analysis).

In the ANOVA analysis, post-hoc analysis was conducted using Scheffe's test.

For comparisons that were significant, the effect size (Cohen's d) was calculated, obtaining values ranging from small (d = 0.15) or less to medium (d = 0.30).

As regards the amount of PA performed, we immediately note how moderate PA shows no relationship of any kind, except for the habit dimension (0.18). It thus appears that this level of physical activity, i.e. when one does only a little movement, is not associated with particular attitudes and strategies, and it is not surprising how the aspects of a habitual nature find a weak but significant relationship. In the case of VPA, the picture changes radically, with intense physical activity showing relationships, not high but significant, with all the dimensions considered by the M-PAC tool. Notably, here as well, it can be observed that the strongest relationship is with the identity dimension (0.51), showing that being protagonists in the field of sports greatly contributes to forming personality both in one’s own eyes and in the eyes of others.

Moving on to the sociographic variables, many significant differences can be observed. Once again, the habit dimension differentiates males and females to the detriment of the latter. In the case of parents’ educational background, there clear differences, with the recurring pattern rewarding the children of parents with high school diplomas or college degrees to the detriment of those without at least one of those qualifications. There is also a widespread presence of significant relationships for adolescents whose parents practice PA, with this characteristic increasing the values of almost all the summary indicators. This driving role is also played by parents who practice physical activity professionally. In this case, however, the differentiation is significant only for the ‘identity’ indicator. The educational background of parents is here, more than any other variable, decisive in all the aspects surveyed. Having parents who practice sports seems to be more directly related to capacity, identity, intention, attitudes and opportunity, while having at least one parent who practices sport professionally affects ‘identity’.

### Social support received in PA

Let us now move on to the second cornerstone of the central part of the questionnaire (panel C) referring to the support received by those who practice PA from the social environment, specifically from family members, friends and coaches, with the latter figure being relevant only for those involved in competitive sports in sports clubs. For each of the three reference figures, the questions ask respondents to evaluate to what extent they receive support in terms of encouragement, sharing in the PA, logistical support provided to help pursue the PA and positive feedback received regarding athletic performance. Here too, for each of the figures, the presence of a one-dimensional construct was verified, obtaining indications of acceptable or good consistencies. The construction of summary indicators, referring to the three different figures, was thus provided.

The indicator referring to the family (Table 5) shows that it provides a fair amount of support (0.54), substantially in line with the support received from friends (0.53), at least in the current period, whereas in the pre-pandemic phase, the support received from friends was considerably lower (0.38 vs 0.50). The support provided by coaches, who are very engaged and supportive, appears to be very positive, especially in the post-pandemic phase (0.71 vs 0.59, + 20%). However, bear in mind that here we are referring only to adolescents involved in competitive sports, and in this case, they rely on coaches not only for technical but also for emotional support.

**Table 5:** Support received in carrying out physical activity (ASAFA Instrument). Average values (AV), standard deviations((SD)) and internal consistency (Crombach's Alpha) of summary section indicators in the present or near future and in the pre-COVID-19 period (n = various).

Sections (Items)	Current/Next 4 weeks		Pre COVID-19	
	Alpha	AV (SD)	Alpha	AV (SD)
Family members (5)	0.67	0.54 (0.21)	0.75	0.50 (0.25)
Friends (5)	0.81	0.53 (0.28)	0.88	0.38 (0.31)
Coaches (5)	0.74	0.71 (0.22)	0.88	0.59 (0.31)

#### Social support, amount of physical activity, and sociographic characteristics

Here we describe framework C of the questionnaire, which aims to find possible links between the summary measures referring to the groups of subjects who provide support to the students and sociographic variables, including those related to the amount of PA reported by the subjects. The summary data referring to the analyses carried out are illustrated in Table 6 and, as is now established practice, in the comments we will limit ourselves to the statistically significant indications.

**Table 6:** Comparisons between the summary indicators by the type of subjects providing support to the subjects evaluated in the current period, amount of physical activity currently performed and the sociographic characteristics of the respondents (n = various).

Item	Source of support		
	Family members	Friends	Coaches
Weekly amount of moderate activity	0.01	0.06	
Weekly amount of vigorous activity	0.22^(^**^)^	0.25^(^**^)^	0.15^(^**^)^
Gender	*		
Female	0.53 (0.21)	0.48 (0.27)	0.71 (0.21)
Male	0.56 (0.21)	0.57 (0.28)	0.70 (0.22)
Educational qualifications of parents/family members	**		
No one has a high school diploma	0.46 (0.18)		
At least one has a high school diploma	0.56 (0.20)		
At least one has a college degree	0.57 (0.21)		
Sports practiced by parent(s)/family members	**		
Yes	0.58 (0.20)		
No	0.45 (0.21)		
Sports practiced professionally	**		
Yes	0.62 (0.22)		
No	0.53 (0.20)		

*One or more significant comparisons at 0.05 (dependent-samples t test; ANOVA analysis).

**One or more significant comparisons at 0.01 (dependent-samples t test; ANOVA analysis).

In the ANOVA analysis, post-hoc analysis was conducted using Scheffe’s test.

For comparisons that were significant, the effect size (Cohen's d) was calculated,

obtaining values ranging from small (d = 0.15) or less to medium (d = 0.30).

There does not appear to be any relationship between moderate PA and the support provided. In fact, this type of activity is often considered a recreational activity and therefore is unlikely to be viewed as an activity requiring support from parents and friends. Intense activities are clearly different. Indeed, here we see a relationship, not strong but nonetheless significant, between the amount of hours dedicated to intense PA and the relative values of the indicators of support received, confirming that increasing commitment requires adequate support.

Having a family member with an educational qualification leads to an increase in the family support indicator. The same heightened support is also found when parents practice PA or do it professionally. Here, we can hypothesize that family engagement in PA reflects a different level of sensitivity to PA on the part of the family members.

By far, the figures who provide the most support, compared to family and friends both in the pre and post COVID-19 phases, are professional coaches. Coach support for sport and PA appears stronger in the post-COVID-19 phase.

### M-PAC indicators and social support

Finally, we investigated a possible correlation between the indicators relating to the SS received and all the indicators related to the dimensions of the M-PAC instrument. The underlying hypothesis is that attitudes and processes regarding PA are also the result of a peaceful and participatory environment of family and friends, from which the young person who practices PA receives help and support. The same is true of the social environment of sports clubs. Indeed, the study of the reciprocal correlations (Table 7) shows a widespread presence of uniformly positive and highly significant relationships (*p* < 0.01). In the case of the current period, the correlation coefficient ranges from a minimum value of 0.09 (very weak relationship) up to a maximum of 0.38, with the highest values referring to the identity dimension of the adolescents. A similar, and even more comforting picture can be observed in the pre-COVID-19 period. Here the correlation coefficients range from a minimum of 0.21 up to a maximum of 0.54, with the highest values also attributable to the identity dimension.

**Table 7:** Correlation coefficients between summary section indicators of support received in carrying out physical activity (ASAFA Instrument) and summary section indicators of the M-PAC Instrument in the present or near future and in the pre-COVID-19 period (n = various).

M-PAC Sections	Affectivity	Instrumentality	Capacity	Opportunity	Intention	Behavioral regulation	Habit	Identity
ASAFA sections								
Current/Next 4 weeks								
Family members	0.26**	0.16**	0.26**	0.24**	0.17**	0.21**	0.25**	0.38**
Friends	0.19**	0.09**	0.14**	0.17**	0.24**	0.30**	0.29**	0.37**
Coaches	0.20**	0.15**	0.23**	0.22**	0.18**	0.24**	0.26**	0.28**
Pre COVID-19								
Family members	0.40**	0.32**	0.41**	0.36**	–	0.39**	0.44**	0.52**
Friends	0.26**	0.21**	0.31**	0.37**	–	0.41**	0.45**	0.49**
Coaches	0.38**	0.37**	0.41**	0.48**	–	0.38**	0.49**	0.54**

**p < 0.01.

Having confirmed the overall pattern of stronger correlations in the pre-Covid period, one wonders what a possible explanation might be. The data are helpful in providing elements supporting the hypothesis that the changes that occurred may have increased adolescents’ independence, leading them to emancipate themselves from the support received in each of the M-PAC framework dimensions. This fact that the adolescent subjects were older in the post-Covid phase may have also contributed to their greater independence.

## Discussion

This study focuses on adolescents’ cognitive processes, behaviors and SS as they relate to PA before and after the pandemic. It contributes to the knowledge of the changes that occurred in some personal attributes and social aspects in relation to PA in adolescents as a consequence of the COVID-19 pandemic. The knowledge of these changes correlated with PA engagement in the post-pandemic phase, clarified their impact, and it can help to formulate appropriate strategies for interventions to promote PA during pandemics and other global crisis.

Increasing values were found in the post-COVID-19 phase for all the cognitive processes and attitudes, in particular, those regarding habits (0.66 vs 0.50, + 32%) and identity (0.70 vs 0.55, + 27%) related to PA. Significant correlations were found between these values and VPA engagement and between the values of the same indicators and SS (*p* < 0.01). The strongest relationship was found with the dimension of identity (r = 0.51; r = 054).

Overall, the results of the survey confirm what is already known in the literature regarding the commitment to PA in relation to the gender of the practitioners^50–52^ and the role played by the parental figures in the choice of PA practicing^53,54^. In particular, males result more in the habit of practicing PA than females. Parents who have a diploma or degree, practice PA and are sports professionals exert an imitative effect and influence their children’s decisions to participate in PA.

The results suggest that pandemic-related restrictions impacted the M-PAC indicators related to PA in adolescents. In general, adolescents now seem willing to adopt more virtuous attitudes and behaviors in PAMV, as evidenced by the values reported in attitudes, skills, opportunities, behaviors, habits and identities in relation to PA. Indeed, on the basis of what the subjects reported, there were increases in all indicators of the M-PAC tool in the post-pandemic phase compared to the pre-pandemic phase. The relative increases are clear and the most conspicuous are found in the dimensions of habit (0.66 vs 0.50, + 32%), identity (0.70 vs 0.55, + 27%), and behavioral regulation (0.57 vs 0.46, + 24%), while the smallest increases are found in instrumental attitudes (0.83 vs 0.72, + 15%) and opportunities (0.68 vs 0.58, + 17%).

The significant relationship found in previous studies^12,13^ between habit and PA in adolescence is confirmed. In fact, in our research, habit appears to be the M-PAC indicator most significantly correlated to the amount of both moderate and vigorous physical activity. The pandemic seems to have changed habit towards PA, and this change seems to have favored PA involvement in the post-Covid phase, although it is a country (Italy) where heavy restrictions have been applied. This result is in line with the results reported by the Schöttl study^8^.

With regard to intention in PA for the immediate future, equally positive values were reported (58.2% of subjects declared a commitment to MVPA for 150′ a week to be extremely probable). Although intentions are not always a strong predictor of behaviors, they are considered weak to moderately related to PA^39^. Perhaps most importantly, intentions are considered a necessary condition for most people to engage in behaviors of participation in PA^55^. They are a necessary but not sufficient condition. Perceived attitudes and abilities, which in the survey show an increase in the post-pandemic phase, also play a role in the formation of intention and should remain stable even in the face of major changes and critical issues.

In our study all the indicators of the M-PAC tool show significant correlations with the amount of vigorous PA. This result confirms the thesis expressed by Cury^17^. Nevertheless, in our case this result demonstrates di be also extendible to adolescents’ PA engagement in the post-pandemic phase.

As regards the amount of PA performed, moderate intensity PA does not appear to show significant relationships with the M-PAC dimensions, except for the dimension of habit (r = 0.18, *p* < 0.01). In the case of vigorous PA, the picture changes radically, with vigorous PA showing significant relationships (*p* < 0.01) with all the dimensions considered by the questionnaire. The strongest relationship was found with the dimension of identity (r = 0.51), demonstrating that being a protagonist in PA contributes greatly to developing one’s personality, both in one’s own eyes and in the eyes of others, in particular, in the case of vigorous PA. Probably many adolescents feel the need to provide for self-identification through PA recognizing themselves as physically active, this occurs to a greater extent after the uncertainties related to the experience of the pandemic, and therefore to a greater extent than in the pre-COVID-19 when they were younger and hadn’t yet experienced the pandemic.

Regarding identity, we find that this indicator is associated with PAMV also in the post-pandemic phase: this result integrates the results of the research developed by Kwan et al.^15^. In our study a significant relationship is found between PA and all the dimensions of the M-PAC instrument, but the strongest relationship occurs with the identity dimension. In fact, as physical activity amount increases the identity values also increase, as shown by the value of the correlation coefficient (0.51).

Another notable finding was the increase in regulated behavior after the COVID-19 pandemic (0.86 vs 0.76). This finding suggests that the restrictions led to the adaptive use of behavioral regulation skills such as action planning and coping, and to plan for and deal with potential or unexpected problems. Since many of the restrictions imposed likely caused disruptions in typical behaviors, adolescents have found themselves forced to strategically formulate and implement new plans for being physically active. Our results suggest that in addition to efforts to help increase intention toward PA, potentially tending to decrease during critical phases, more emphasis should be placed on helping adolescents regulate their behaviors through self-monitoring, setting goals, planning and managing major changes in order to become more resilient and bridge the intention-action gap, regardless of ever-changing circumstances.

As a confirmation and as an integration of the same study by Kwan et al.^15^, we found that coping is significantly associated with PAV also in the post-pandemic phase. Indeed, in our study regulated behavior after the COVID-19 pandemic is shown to be related to the amount of PAV, suggesting that PAV represented a tool for adolescents to acquire adaptive use of behavioral regulation skills such as action planning and coping.

Finally, our analysis of the interrelationship between the indicators relating to SS and the attitudes and processes regarding PA showed positive and highly significant correlations (*p* < 0.01) across the board. In the case of the current period, the correlation coefficient ranges from a minimum value of 0.09 (very weak relationship) up to a maximum of 0.38, with the highest values referring to the identity dimension of adolescents. The same pattern was found in the pre-COVID-19 period. Here the correlation coefficients range from a minimum of 0.21 up to a maximum of 0.54, with the highest values again attributable to the identity dimension.

The results of this study relating to SS confirm the importance it has for maintaining involvement in PA during the pandemic and in contexts of social restrictions, as affirmed by Ng et al.^20^ and by Hailey^18^. In our study these results prove to be also extendible to the post-pandemic phase. Here we found a not intense but still significant relationship between the social support received and the amount of PAV practiced. SS from different sources appears significantly correlated with the amount of PAV, with the following correlation coefficients: family (0.22), peer group (0.25) and coaches (0.15).

In our study a positive correlation is found between all M-PAC indicators and SS, but this correlation is less strong in the post COVID-19 phase compared to the pre COVID-19 phase. An explanation here requires a consideration of other variables such as students’ increased age in the transition between the two periods considered and a possible increased request for autonomy and emancipation from the significative others.

Future research should focus on the whole spectrum of personal and social attributes that can influence physical activity engagement during pandemics, so as to prevent post-pandemic declines of PA with the already known effects on adolescents’ health and well-being in their transition to adulthood.

While this study presents new findings, it has several limitations that need to be acknowledged. The first limitation is of a temporal nature, as we could not capture the initial changes in PA behaviors and cognitions during the onset of the COVID-19 pandemic. By the time students were administered the questionnaire in April–May 2023, they may have already adopted strategies to better cope with the unique challenges of the pandemic years. Second, PA-related behaviors were measured using self-report assessments and are therefore susceptible to overreporting due to social desirability biases^56^. However, the use of the IPAQ-SF as a self-report measure for MVPA is a strength, considering that it is one of the most commonly used self-report questionnaires for measuring PA in the literature, allowing for comparisons between studies. Finally, given that this study took place in a single geographic area, these findings may not apply to other regions or countries that may have implemented different COVID-19 pandemic restrictions.

## Data Availability

Figshare.com. Link: https://figshare.com/articles/dataset/Mario_Corsi_Data/23628585. Contains: DAT. CSV [Dati Indagine (survey data)]. DATA READ ME.Txt [Istruzioni (Instructions)].

## References

[1] Kelly AL, Erickson K, Turnnidge J (2020). Youth sport in the time of COVID-19: Considerations for researchers and practitioners. Manag. Sport Leis..

[2] Lu C, Chi X, Liang K, Chen S-T, Huang L, Guo T, Jiao C, Yu Q, Veronese N, Soares FC (2020). Moving more and sitting less as healthy lifestyle behaviors are protective factors for insomnia, depression, and anxiety among adolescents during the COVID-19 pandemic. Psychol. Res. Behav. Manag..

[3] Minuto N, Bassi M, Montobbio C, Vinci F, Mercuri C, Perri FN, Cabri M, Calevo MG, D’Annunzio G, Maghnie M (2021). The effect of lockdown and physical activity on glycemic control in italian children and young patients with type 1 diabetes. Front. Endocrinol..

[4] Frömel K, Groffik D, Valach P, Šafář M, Mitáš J (2022). The impact of distance education during the COVID-19 pandemic on physical activity and well-being of Czech and Polish Adolescents. J. Sch. Health.

[5] Bozzola E, Barni S, Ficari A, Villani A (2023). Physical Activity in the COVID-19 era and its impact on Adolescents’ Well-Being. Int. J. Environ. Res. Public Health.

[6] Janssen I, LeBlanc AG (2010). Systematic review of the health benefits of physical activity and fitness in school-aged children and youth. Int. J. Behav. Nutr. Phys. Act..

[7] Granger E, Di Nardo F, Harrison H, Patterson L, Holmes R, Arpana V (2017). A systematic review of the relationship of physical activity and health status in adolescents. Eur. J. Public Health.

[8] Schöttl S. E., Schnitzer M., Savoia L. & Kopp, M. Physical activity behavior during and after COVID-19 stay-at-home orders. A Longitudinal Study in the Austrian, German, and Italian Alps. *Front. Public Health*. Sec. Planetary Health Vol. 10 – 2022 (2022). 10.3389/fpubh.2022.901763 10.3389/fpubh.2022.901763 PMC9194442 35712287

[9] IOC (International Olympic Committee). Available at: https://olympics.com/ioc/overview; https://olympics.com/ioc/news/ioc-emphasises-importance-of-sport-in-covid-19-recovery-efforts-at-european-commission-conference (Accessed on 06//2023)

[10] Chulvi-Medrano I, Thomas E, Padua E (2022). Physical exercise for health and performance post-pandemic COVID-19 Era, a renewed emphasis on public health. Int. J. Environ. Res. Public Health.

[11] Park AH, Zhong S, Yang H, Jeong J, Lee C (2022). Impact of COVID-19 on physical activity: A rapid review. J. Glob. Health.

[12] Gardner B, Arden MA, Brown D, Eves FF, Green J, Hamilton K (2021). Developing Habit-based health behaviour change interventions: Twenty-one questions to guide future research. Psychol. Health.

[13] Ma H, Wang A, Pei R, Piao M (2023). Effects of habit formation interventions on physical activity habit strength: Meta-analysis and meta-regression. Int. J. Behav. Nutr. Phys. Act..

[14] Rhodes RE, Liu S, Lithopoulos A, Zhang CQ, Garcia-Barrera MA (2020). Correlates of perceived physical activity transitions during the COVID-19 pandemic among Canadian adults. Appl. Psychol. Health Well Being.

[15] Kwan MY, Brown DM, Dutta P, Haider I, Cairney J, Rhodes RE (2021). Application of the Multi-Process Action control model to predict physical activity during late adolescence. J. Sport Exerc. Psychol..

[16] Jiao WY, Wang LN, Liu J, Fang SF, Jiao FY, Pettoello-Mantovani M, Somekh E (2020). Behavioral and emotional disorders in children during the COVID-19 epidemic. J. Pediatr..

[17] Cury F (2002). Perceptions of competence, implicit theory of ability, perception of motivational climate, and achievement goals: A test of the trichotomous conceptualization of endorsement of achievement motivation in the physical education setting. Percept. Mot. Skills.

[18] Hailey V, Fisher A, Hamer M, Fancourt D (2023). Perceived social support and sustained physical activity during the COVID-19 pandemic. Int. J. Behav. Med..

[19] Elliott S, Drummond MJ, Prichard I (2021). Understanding the impact of COVID-19 on youth sport in australia and consequences for future participation and retention. BMC Public Health.

[20] Ng K, Cooper J, McHale F, Clifford J, Woods C (2020). Barriers and facilitators to changes in adolescent physical activity during COVID-19. BMJ Open Sport Exerc. Med..

[21] Moore SA, Faulkner G, Rhodes RE, Brussoni M, Chulak-Bozzer T, Ferguson LJ (2020). Impact of the COVID-19 virus outbreak on movement and play behaviours of Canadian children and youth: a national survey. Int. J. Behav. Nutr. Phys. Act..

[22] Tison GH, Avram R, Kuhar P, Abreau S, Marcus GM, Pletcher MJ (2020). Worldwide effect of COVID-19 on physical activity: a descriptive study. Ann. Intern. Med..

[23] Chaffee BW, Cheng J, Couch ET, Hoeft KS, Halpern-Felsher B (2021). Adolescents’ substance use and physical activity before and during the COVID-19 pandemic. JAMA Pediatr..

[24] Stockwell S, Trott M, Tully M, Shin J, Barnett Y, Butler L (2021). Changes in physical activity and sedentary behaviours frombefore to during the COVID-19 pandemic lockdown: A systematic review. BMJ Open Sport Exerc. Med..

[25] Wunsch K, Kienberger K, Niessner C (2022). Changes in physical activity patterns due to the COVID-19 pandemic: A systematic review and meta-analysis. Int. J. Environ. Res. Public Health.

[26] Amatori S, Donati Zeppa S, Preti A, Gervasi M, Gobbi E, Ferrini F, Rocchi MBL, Baldari C, Perroni F, Piccoli G (2020). Dietary habits and psychological states during COVID-19 home isolation in italian college students: The role of physical exercise. Nutrients.

[27] Aucejo EM, French J, Ugalde Araya MP, Zafar B (2020). The impact of COVID-19 on student experiences and expectations: Evidence from a survey. J. Public Econ..

[28] Son C, Hegde S, Smith A, Wang X, Sasangohar F (2020). Effects of COVID-19 on college students' mental health in the United States: Interview survey study. J. Med. Internet Res..

[29] Zhang Y, Zhang H, Ma X, Di Q (2020). Mental health problems during the COVID-19 pandemics and the mitigation effects of exercise: A longitudinal study of college students in China. Int. J. Environ. Res. Public Health.

[30] Du C, Zan MCH, Cho MJ, Fenton JI, Hsiao PY, Hsiao R, Keaver L, Lai CC, Lee H, Ludy MJ, Shen W, Swee WCS, Thrivikraman J, Tseng KW, Tseng WC, Almotwa J, Feldpausch CE, Folk SYL, Gadd S, Wang L, Wang W, Zhang X, Tucker RM (2021). Health behaviors of higher education students from 7 countries: Poorer sleep quality during the COVID-19 pandemic predicts higher dietary risk. Clocks Sleep.

[31] Warburton DE, Nicol CW, Bredin SS (2006). Health benefits of physical activity: The evidence. Can. Med. Assoc. J..

[32] Donnelly JE, Hillman CH, Castelli D, Etnier JL, Lee S, Tomporowski P (2016). Physical activity, fitness, cognitive function, and academic achievement in children: A systematic review. Med. Sci. Sport. Exerc..

[33] Biddle SJ, Ciaccioni S, Thomas G, Vergeer I (2019). Physical activity and mental health in children and adolescents: An updated review of reviews and an analysis of causality. Psychol. Sport Exerc..

[34] Tammelin T, Laitinen J, Näyhä S (2004). Change in the level of physical activity from adolescence into adulthood and obesity at the age of 31 years. Int. J. Obes..

[35] Kwon S, Janz KF, Letuchy EM, Burns TL, Levy SM (2015). Developmental trajectories of physical activity, sports, and television viewing during childhood to young adulthood: IOWA bone development study. JAMA Pediatr..

[36] Liu S, Husband C, La H, Juba M, Loucks R, Harrison A (2019). Development of a self-guided web-based intervention to promote physical activity using the multi-process action control framework. Internet Interv..

[37] Craig CL, Marshall AL, Sjöström M, Bauman AE, Booth ML, Ainsworth BE, Pratt M, Ekelund U, Yngve A, Sallis JF, Oja P (2003). International Physical Activity Questionnaire: 12-Country reliability and validity. Med. Sci. Sport. Exerc..

[38] Mannocci A, Di Thiene D, Del Cimmuto A, Masala D, Boccia A, De Vito E, La Torre G (2010). International physical activity questionnaire: Validation and assessment in an Italian sample. Ital. J. Public Health.

[39] Rhodes, R. E. The evolving understanding of physical activity behavior: a multi-process action control approach, in advances in motivation science, 4th edn. (ed by Elliot, A. J.) 171–205 (Open Access: Science Direct, 2017). 10.3389/fspor.2022.895097

[40] Rhodes RE (2021). Multi-process action control in physical activity: A primer. Front. Psychol. Sect. Health Psychol..

[41] Rhodes RE, La H, Quinlan A, Grant S, Englert C, Taylor I (2021). Enacting physical activity intention: A multi-process action control approach. Motivation and Self-Regulation in Sport and Exercise.

[42] Vallerand JR, Rhodes RE, Walker GJ, Courneya KS (2017). Correlates of meeting the combined and Independent Aerobic and Strength Exercise Guidelines in Hematologic Cancer Survivors. Int. J. Behav. Nutr. Phys. Act..

[43] Rhodes RE, Quinlan A, Naylor PJ, Warburton DE, Blanchard CM (2020). Predicting personal physical activity of parents during participation in a family intervention targeting their children. J. Behav. Med..

[44] de Farias C, Mendonça G, Florindo AA, Gomes de Barro MV (2014). Reliability and validity of a physical activity social support assessment scale in adolescents—ASAFA Scale. Rev. Bras. Epidemiol..

[45] Mendonça G, Cheng LA, Mélo EN, Farias Junior JC (2014). Physical activity and social support in adolescent: A systematic review. Health Educ. Res..

[46] Yao CA, Rhodes RE (2015). Parental correlates in child and adolescence physical activity: A meta-analysis. Int. J. Behav. Nutr. Phys. Act..

[47] Laird Y, Fawkner S, Kelly P, Mcnamee L, Niven A (2016). The role of social support on physical activity behavior in adolescent girls: A systematic review and meta-analysis. Int. J. Behav. Nutr. Phys. Act..

[48] Duncan SC, Duncan TE, Strycker LA (2005). Sources and types of social support in youth physical activity. Health Psychol..

[49] Van der Horst K, Paw MJCA, Twisk JWR, Van Mechelen W (2007). A brief review on correlates of physical activity and sedentariness in youth. Med. Sci. Sport. Exerc..

[50] Chinurum JN, OgunjImi LO, O’Neill CB (2014). Gender and sports in contemporary society. J. Educ. Soc. Res..

[51] Lagaert S, Roose H (2016). The gender gap in sport event attendance in Europe: The impact of macro-level gender equality. Int. Rev. Sociol. Sport.

[52] European Commission (EU) 2022. Special Eurobarometer 525 Sport and Physical Activity (V°).

[53] Henriksen PW, Ingholt L, Rasmussen M, Holstein BE (2015). Physical activity among adolescents: The role of various kinds of parental support. Scand. J. Med. Sci. Sport..

[54] Khan SR, Uddin R, Mandic S, Khan A (2020). Parental and peer support are associated with physical activity in adolescents: Evidence from 74 countries. Int. J. Environ. Res. Public Health.

[55] Rhodes RE, de Bruijn GJ (2013). How big is the physical activity intention behavior gap? A meta-analysis using the action control framework. Br. J. Health Psychol..

[56] Prince SA, Adamo KB, Hamel ME, Hardt J, Gorber SC, Tremblay MA (2008). Comparison of direct versus self-report measures for assessing physical activity in adults: A systematic review. Int. J. Behav. Nutr. Phys. Act..

